# Supplementary material for: Integrated cooling (i-Cool) textile of heat conduction and sweat transportation for personal perspiration management
Source: Nat Commun. 2021 Oct 21;12:6122. doi: 10.1038/s41467-021-26384-8 (PMC8531342; doi:10.1038/s41467-021-26384-8)
Supplement: Supplementary file 1 — Supplementary information [file 41467_2021_26384_MOESM1_ESM.pdf]

Supplementary Information for

Integrated Cooling (i-Cool) Textile of Heat Conduction and Sweat Transportation for Personal Perspiration Management

Yucan Peng[1†], Wei Li[2,3†], Bofei Liu[1], Weiliang Jin[2], Joseph Schaadt[4,5], Jing Tang[1], Guangmin Zhou[1], Guanyang Wang[6], Jiawei Zhou[1], Chi Zhang[7], Yangying Zhu[1], Wenxiao Huang[1], Tong Wu[1], Kenneth E. Goodson[7], Chris Dame[4,5], Ravi Prasher[4,5], Shanhui Fan[2] & Yi Cui[1,8*]

[1]Department of Materials Science and Engineering, Stanford University, Stanford, CA 94305, USA.

[2]E. L. Ginzton Laboratory, Department of Electrical Engineering, Stanford University, Stanford, CA 94305, USA.

[3]GPL Photonics Lab, State Key Laboratory of Applied Optics, Changchun Institute of Optics, Fine Mechanics and Physics, Chinese Academy of Sciences, Changchun, 130033, China.

[4]Department of Mechanical Engineering, University of California, Berkeley, CA 94720, USA

[5]Energy Technologies Area, Lawrence Berkeley National Laboratory, 1 Cyclotron Road, Berkeley, CA 94720, USA

[6]Department of Mathematics, Stanford University, Stanford, CA 94305, USA

[7]Department of Mechanical Engineering, Stanford University, Stanford, CA 94305, USA.

[8]Stanford Institute for Materials and Energy Sciences, SLAC National Accelerator Laboratory, 2575 Sand Hill Road, Menlo Park, CA 94025, USA.

[†]These authors contributed equally to this work

***Corresponding author: Yi Cui (yicui@stanford.edu)**

Supplementary Note 1. Water vapour transmission property for textiles

Water vapour transmission, namely breathability[1,2], is an essential property for textiles for ensuring wearing comfort. The human body releases water vapour all the time, no matter whether sensible perspiration happens. At a dry state, water vapour loss from human body (insensible perspiration) accounts for about 20 percent of the total heat dissipation[3,4]. As human body heat load increases, human body starts to secrete sweat to release excessive heat. During a slight perspiration, sweat can almost be evaporated fast on the skin directly and nearly only water vapour passes through the textile. Thus, decent water vapour transmission rate of textiles is necessary for achieving good cooling effect in these situations. We characterized the water vapour transmission rate of textiles using the upright cup method (ASTM E96). As shown in Supplementary Fig. 1a, the i-Cool (Cu) textile shows comparable water vapour transmission rate to other commercial textiles such as cotton, which have been widely accepted as a good water vapour permeable textiles. We also performed the water vapour thermal measurement, in which sweat was evaporated directly on the simulated skin surface and only water vapour passes through textiles (Supplementary Fig. 1b). The measured skin temperature was overall negatively correlated with the water vapour transmission rate, which indicates the higher water vapour transmission rate can generally achieve better cooling effect for the skin in a vapour-only scenario, even though water vapour condensation and textile dry thermal resistance can also influence the measured skin temperature. Moreover, measurements of evaporative resistance based on ISO11092:2014/ASTM 1868-17 were performed, and the apparatus schematic and results are exhibited in Supplementary Fig. 2. All of the tested textiles can be considered as very good in breathability according to the Hohenstein comfort rating[5]. The above test results indicate that the i-Cool (Cu) textile and other commercial textiles exhibit decent water vapour transmission ability so that they all can perform well in human body's mild scenarios (dry state and slight perspiration). However, how textiles handle the liquid sweat and releases its evaporative cooling power should be emphasized in the moderate/profuse perspiration scenario.

Supplementary Note 2. Thermal resistance measurement with cut bar method

The cut bar method used in measuring the thermal resistance of these sheet-like samples is a standard technique adapted from the ASTM-5470 standard[6], using an apparatus described elsewhere[7]. As shown in Supplementary Fig. 8, the measurement principle relies on uniform steady

state 1-D heat conduction to measure the heat flow and temperature drop across a sample (either a single sheet or a stack of multiple sheets). In this setup, eight thermocouples are inserted into the center of two 1 inch ×1 inch copper reference bars to measure the temperature profiles $T(z)$ along the top and bottom bar. A resistance heater generates a heat flux q which flows through the top bar followed by the sample and then the bottom bar after which the heat is dissipated into a large heat sink. The temperature profiles of the top and bottom copper bars are then used to determine both the heat flux q through the sample and the temperature drop across the sample stack, $\Delta T_s = T_H - T_L$. The entire apparatus (top bar, sample, bottom bar) is wrapped in thermal insulation. A modest pressure of approximately 15 psi was applied at the top bar to reduce contact resistance, and no thermal grease was used due to the material porosity. At these pressures the i-Cool does not compress visibly while the other textiles do. The thermal resistances of uncompressed textiles would be even larger than those measured here and reported in Fig. 2d of the main text, making the relative performance of i-Cool (Cu) even more impressive. In operation, the temperature profile $T(z)$ of the thermocouples is measured and T_H and T_L are determined by extrapolating the measured $T(z)$ profiles to the respective bars' surfaces. Also, using the known thermal conductivity of copper k_{Cu} the heat flux passing through the copper bars is calculated from Fourier's law:

$$q = -k_{\mathrm{Cu}} \frac{dT}{dz} \quad (1)$$

The q calculated for upper and lower bars can differ slightly due to minor heat losses from the upper bar[7]; for this reason, we use the q calculated for the lower bar for the rest of the analysis. As mentioned previously, the temperature profiles along the two copper reference bars are used to calculate the temperature drop between bar surfaces by linear extrapolation. Finally, the total thermal resistance from T_H to T_L, including the sample(s) plus contacts, is then calculated from

$$R_{\mathrm{TOT}} = \frac{\Delta T_s}{q} \quad (2)$$

The diagram in the red dash box in Supplementary Fig. 8 illustrates the thermal resistances for a typical stack of N = 3 samples, where R_{s} is the thermal resistance of a single sample sheet, $R_{\mathrm{c,\,ss}}$ is the thermal contact resistance between adjacent samples, and $R_{\mathrm{c,sb}}$ is the thermal contact resistance between a sample and a copper reference bar. For arbitrary N, by adding these resistances in series it is clear that

$$R_{\mathrm{TOT}} = N\left(R_{\mathrm{s}} + R_{\mathrm{c,\,ss}}\right) + \left(2R_{\mathrm{c,sb}} - R_{\mathrm{c,ss}}\right) \tag{3}$$

Plotted on R_{TOT} vs. N axes, this equation takes the form of a straight line. The slope $\left(R_{\mathrm{s}} + R_{\mathrm{c,\,ss}}\right)$ contains both the thermal resistance of the sample and the thermal contact resistance between samples, and the intercept $\left(2R_{\mathrm{c,sb}} - R_{\mathrm{c,ss}}\right)$ contains both types of thermal contact resistance. Because fitting the line gives only two pieces of information (slope and intercept) but there are three unknowns (R_{s}, $R_{\mathrm{c,\,ss}}$, and $R_{\mathrm{c,sb}}$), it is not possible to isolate R_{s} separately from the effect of $R_{\mathrm{c,\,ss}}$ in this measurement.

Typical measurement results for $R_{\mathrm{TOT}}(N)$ for the i-Cool (Cu) samples gives $R_{\mathrm{s}} + R_{\mathrm{c,\,ss}} =$ 0.0001895 $m^2K/W \pm 9\%$. Because $R_{\mathrm{c,\,ss}}$ cannot be measured independently, we instead roughly estimate its possible effect by considering a range of reference values for solid-solid interfaces at modest pressures[8], which are typically in the range $R_{\mathrm{c,\,ss}}$ between ~1×10^{-5} to ~1×10^{-4} m^2K/W. Subtracting this range of estimated $R_{\mathrm{c,\,ss}}$ values from the measured $R_{\mathrm{s}} + R_{\mathrm{c,\,ss}}$ gives a bounding range on R_{s} of between 0.0000895 m^2K/W to 0.0001795 m^2K/W.

To interpret the heat conduction pathways in the i-Cool (Cu), we developed a simple thermal resistor model depicted in Supplementary Fig. 9a. In this circuit, there are two types of parallel pathways. The first type of pathway is columns of pure polymer, of length (l_1 + l_2), thermal conductivity k_{p}, and total cross-sectional area ϕA, where $\phi \approx 0.32$ is the porosity of the copper foil and A is the total area of the sample. Similarly, the second type of pathway is columns of (copper + polymer), where each such column has l_1 of copper in series with l_2 of polymer, and the total cross sectional area is $(1-\phi)A$. Note that A will cancel out at the end of the calculation since we are presenting all thermal resistances on an area-normalized basis (SI units: $m^2\cdot K/W$).

Solving the resistor network and rearranging, the thermal resistance of the sample per unit area [m^2K/W] is

$$R_{\mathrm{s}} = \left(\frac{k_{\mathrm{p}}\phi}{l_1 + l_2} + \frac{1}{\left(\frac{l_1}{k_{\mathrm{Cu}}(1-\phi)} + \frac{l_2}{k_{\mathrm{p}}(1-\phi)}\right)}\right)^{-1} \tag{4}$$

where k_{Cu} and k_{p} are the thermal conductivity of copper and nylon 6 respectively, l_1 and l_2 are the thickness of copper and nylon 6 nanofibres respectively, and ϕ is the porosity of the copper foil. The R_{s} can be calculated by setting the parameter values: k_{Cu} = 400 W/(m·K), k_{p} = 0.28

W/(m·K) (midpoint of representative literature values)[9-11], l_1 = 25 μm, l_2 = 20 μm, and ϕ = 0.32. Using these values, the calculated R_s is 8.7×10^{-5} m^2·K/W. This model value is quite close to the low end (8.95×10^{-5} m^2·K/W) of the range of R_s as determined from the measurements combined with literature estimates for $R_{\mathrm{c,\,ss}}$. Due to the high thermal conductivity of copper, changes to l_1 cause only a minor change to the overall thermal resistance, which indicates there is large flexibility for heat conductive matrix thickness selection. As shown in Supplementary Fig. 9b, increasing the Cu thickness (l_1) from 25 μm to 1000 μm (40 times) only increases the thermal resistance from 8.7×10^{-5} m^2·K/W to 1.08×10^{-4} m^2·K/W (~24% increase).

Supplementary Note 3. Sweat evaporative cooling efficiency

In the steady-state evaporation test, the measured power density (q) of artificial skin can be considered as the total heat loss from two parts: non-evaporation heat loss ($q_{\mathrm{non-evap}}$, including conduction, radiation and convection) and evaporative heat loss due to water vaporization (q_{evap}), as follows.

$$q = q_{\mathrm{non-evap}} + q_{\mathrm{evap}} \quad (5)$$

Here, q_{evap} can be described as

$$q_{\mathrm{evap}} = \frac{\eta \cdot v \cdot L_{\mathrm{water}}}{A} \quad (6)$$

Where η means sweat evaporative cooling efficiency (unitless), v is the evaporation rate, L_{water} is the latent heat of water vaporization at 35 °C (2448 J/g)[12], and A is the textile sample area (i.e. artificial skin area). The physical meaning of η is that the energy taken away by sweat vaporization within the clothes is usually not totally from human body. The sweat evaporation may absorb heat from clothes or environment instead of the human body[13-15]. Therefore, the index η is utilized to describe the actual cooling effect of sweat evaporation. In some literature, $L_{\mathrm{water-eff}}$ was used, rather than η, to describe the same physical mechanism (then, $q_{\mathrm{evap}} = \frac{v \cdot L_{\mathrm{water-eff}}}{A}$)[14].

Employing equation (5) and (6), we can obtain

$$\frac{dq}{dv} = \frac{dq_{\mathrm{evap}}}{dv} + \frac{dq_{\mathrm{non-evap}}}{dv} \quad (7)$$

i.e. $$\frac{dq}{dv} = \frac{L_{\text{water}}}{A}\frac{d(\eta\cdot v)}{dv} + \frac{dq_{\text{non-evap}}}{dv} \tag{8}$$

i.e. $$\frac{dq}{dv} = \frac{L_{\text{water}}}{A}\left(v\cdot\frac{d\eta}{dv} + \eta\right) + \frac{dq_{\text{non-evap}}}{dv} \tag{9}$$

Equation (9) is the rigorous differential equation of q. $\frac{dq}{dv}$ can be derived by fitting the curve of q versus v and calculating its gradient. If $\frac{d\eta}{dv}$ and $\frac{dq_{\text{non-evap}}}{dv}$ are known, accurate η can be obtained at every v. However, it is hard to do so, which at least requires very precise separation of wet conduction and heat pipe heat loss from real evaporative heat loss[15]. Thereby, we simplified equation (9) to calculate η.

In a certain range, $\bar{\eta}$ can be used to represent η, considering $\frac{d\eta}{dv}$ as nearly zero. If $\frac{dq_{\text{non-evap}}}{dv}$ was assumed to be negligible, equation (9) was turned into

$$\frac{dq}{dv} = \frac{L_{\text{water}}}{A}\cdot\bar{\eta} \tag{10}$$

Accordingly, the estimated sweat evaporative cooling efficiency was calculated and exhibited in Supplementary Fig. 13. It is worthwhile to mention that the assumptions are more valid when water mass gain (i.e. evaporation rate in this test) is in the relatively high range, for the same textile. The non-evaporative heat loss change rate in this range should be smaller (closer to 0) than that in the initial dry-to-wet stage. In other words, the estimated sweat evaporative cooling efficiency numbers at 1.1 mL/h, 0.9 mL/h and 0.7 mL/h are considered to be obtained with more valid assumptions.

Supplementary Note 4. Water outflow from a perforated hydrophilic sheet

For a perforated hydrophilic sheet, water can be squeezed out from the heater surface easily, however, the requirement of uniform water outflow is challenging for such perforated membrane design. As displayed in Supplementary Fig. 14a, water cannot outflow uniformly from each pore (diameter at 200 µm) on the surface, even at an ultrahigh flow rate, not only because it is much easier for water to overcome the Laplace pressure at several pores firstly rather than at every pore simultaneously, but also due to the complexity involving fluid dynamics and friction between water and device. Moreover, much larger pores (diameter at 3 mm) were still not able to facilitate

the uniform water outflow (Supplementary Fig. 14b), which furthermore indicates that the design of perforated membrane as skin surface is not feasible enough to provide uniform perspiration condition.

Supplementary Note 5. Fabrication of the Janus-type wicking layer with limited water outlets

We fabricated a Janus-type wicking layer with limited water outlets and added it onto the perforated heater layer as the skin surface to realize uniform perspiration from each artificial sweat pore. To fabricate it, a mask was placed on the wicking layer, then diluted polydimethylsiloxane (PDMS) solution was sprayed on the masked wicking layer. After removing the mask, drying and curing, the uncovered top surface of the wicking layer was modified to be hydrophobic (Supplementary Fig. 15). As illustrated in the red dash box in Fig. 4c, water can diffuse into the unmodified bottom layer with strong wicking ability and be transported to the top surface, while the hydrophobic "baffles" on the top surface will confine water outflow to the unmodified hydrophilic locations. Accordingly, water wicked from the bottom can flow out only from the limited water outlets uniformly to mimic human body perspiration situation (Supplementary Fig. 16).

Supplementary Note 6. Experimental reliability and repeatability of the artificial sweating skin test.

We summarized the skin temperature data of multiple-time measurements. The data distribution histograms are exhibited in Supplementary Fig. 18. Supplementary Figure 18a shows the data distribution of various samples, which clearly demonstrates the significant difference between the i-Cool (Cu) and the conventional textiles. Welch's t-test was also performed to validate the statistical significance ($p < 0.001$). This demonstrates the experimental reliability. Data distribution histogram of multiple tests for the i-Cool (Cu) textile is displayed in Supplementary Fig. 18b, indicating the good repeatability of the measurements.

Supplementary Note 7. Artificial sweating skin test for cotton samples

To eliminate the effect of mass density (or thickness) of textile samples, we tested cotton samples of different area mass density on the artificial sweating skin platform with a feedback control loop. Cotton samples of four different area mass density were prepared by stripping down nonwoven cotton sheets: 26.5 g/m^2, 32 g/m^2, 76 g/m^2, 132 g/m^2. Because the thickness of cotton is hard to accurately measure, we used area mass density to differentiate them. The i-Cool (Cu) textile sample is around 107.7 g/m^2. The 132 g/m^2 sample shows comparable skin temperature to the cotton textile used in the main text (135 g/m^2), thus we assumed the structure difference (knitted vs. nonwoven) can be ignored. The cotton sample of 26.5 g/m^2 was very thin and had no reasonable visible opacity for practical use (Supplementary Figure 20a). We expanded the test range to such thin cotton sample in order to push the effect of area mass density to a limit. It turned out that even such thin cotton sample with extremely low area mass density still cannot achieve comparable evaporative cooling effect to i-Cool (Cu) textile sample (Supplementary Fig. 20b), which exhibited around 1.5 °C higher skin temperature than the i-Cool (Cu) textile. This testifies to the superiority of the i-Cool functional structure design.

Supplementary Note 8. Parameters variation in the i-Cool (Cu) textile

The average skin temperature for the i-Cool (Cu) textile sample in the previous experiment (Fig. 3d) is used as the benchmark, and we performed experiments under the same condition. Firstly, we investigated the thickness influence on cooling effect. Due to the dominant thermal conductivity of Cu, changing its thickness shows slight impact on the performance of the functional structure (Supplementary Fig. 22a). The thickness change of the heat conductive matrix will show negligible effect on the final thermal resistance (Supplementary Fig. 9b). The influence of thermal conductive matrix thickness change on the water transport process should be the dominant factor affecting the resultant cooling performance. Secondly, textile samples containing heat conductive matrix with different thermal conductivity but the same other parameters were studied, as exhibited in Supplementary Fig. 22b. It shows that material with thermal conductivity of the same order of Cu's can lead to comparable skin temperature to i-Cool (Cu). However, low thermal conductivity materials would result in high skin temperature even though the water transport structure was still remained. Furthermore, we varied the pore area ratio on the heat

conductive matrix which referred to Cu matrix here to probe its effect. As shown in Supplementary Fig. 22c, it seems that the pore area ratio in a certain range brings about a negligible influence on cooling performance, but the cooling effect declines when the pore area ratio attains some extent. This is probably because of the reduction of the heat conductive component, which authenticates the structure advantage of the i-Cool textile again. Moreover, the area mass density of nylon 6 nanofibres was altered (Supplementary Fig. 22d). The experimental results indicate that there exists a trade-off in the nylon 6 nanofibres area mass density choice. Decreasing its mass density suggests the thermal conduction in the nylon 6 nanofibres can be better, however its water transport and evaporation area may be negatively influenced thus evaporative cooling effect can be depressed, and vice versa. Therefore, nylon 6 nanofibres with area mass density in the optimal extent is preferred in designing i-Cool textile for personal perspiration management.

Supplementary Note 9. Thermal simulation for actual human body

To model the thermal impacts of the textiles on human body, we set up a coupled heat and mass transfer equation, based on the previously reported models[12, 16-20]. In our simulation, we modeled a human body consisting of passive system and control system. The passive system includes human body tissue sub-system, the circulatory sub-system, and the respiratory sub-system. The control system has vasomotor, sudomotor and metabolic functions, which is for human body thermal responses. In the model, the human body is represented by two concentric shells: core and skin. Above the skin, the clothing forms another layer. The outside of the clothing is ambient air. The schematic of human-clothing-environment system is shown in Supplementary Fig. 23a. The body core, skin, clothing and ambient interact with each other via the blood circulation, respiration, perspiration and evaporation, heat convection and radiation, etc. This model considers the coupled heat and mass transfer across body core, skin, inner and outer surface of textile, as well as the human body dynamic thermal responses such as metabolic heat generation, blood circulation, and perspiration[16].

The thermal model consists of both heat and mass transfer process, considering a four-node system consisting of body core, skin, inner surface of textiles, and outer surface of textiles, with their temperatures represented as T_c, T_s, T_2, T_1, respectively. In the heat transfer part, we consider the thermal balance at each node, starting from the body core:

$$\frac{m_c C_c}{A}\frac{\partial T_c}{\partial t} = M(T_s, T_c) - C_{res}(M, T_a) - E_{res}(M, T_a) - H_b(T_c, T_s) \quad (11)$$

Where m_c is the mass of the body core, A is the area of the body core, C_c is specific heat of body core, $M(T_s, T_c)$ is the metabolic heat production, which equals to the rate of metabolic energy expenditure (M_0) subtracting rate of external work (W). M is the sum of three sources: basal metabolism (M_0), voluntary physical activity (W), and shivering. $C_{res}(M, T_a)$ and $E_{res}(M, T_a)$ are the convective and evaporative heat loss from respiration respectively, and $H_b(T_c, T_s)$ is the heat transfer from body core to skin via blood circulation. C_{res}, E_{res}, H_b will dynamically response to body temperature[17-20]. The heat transfer coefficients are defined as shown in the table.

In the skin layer:

$$d_s \rho_s C_s \frac{\partial T_s}{\partial t} = M_{s,0} + H_b(T_c, T_s) - E_{sw}(T_c, T_s) - E_{diff}(T_s, P_a) - h_{ti}(T_s - T_2) \quad (12)$$

Where d_s, ρ_s, C_s are the thickness, density, and specific heat of the skin layer, respectively. $M_{s,0}$ is the torso skin basal metabolism rate, which is 1.87 W/m (ref. 18 and 19). The skin layer gain heat from body core through blood circulation $H_b(T_c, T_s)$. $E_{sw}(T_c, T_s)$ is heat loss by sweat evaporation, which is calculated by evaporation coefficient (α) * sweat evaporative cooling efficiency (η) * latent heat of water vaporization (L) * sweating rate (regsw). α means the fraction of sweat that is evaporated. η means the fraction of heat taken exactly from the skin by water vaporization. $E_{diff}(T_s, P_a)$ is the heat loss by water vapor diffusing through the skin layer. $h_{ti}(T_s - T_2)$ is the heat transfer from the skin layer to the inner surface of the textile, where h_{ti} is the combined heat transfer coefficient in the microclimate between the skin layer and inner surface of the textile. Here, the adopted h_{ti} values are estimated from additional measurements of each textile on a dry hot plate like Fig. 2e of the main text, which includes microclimate/contact resistance between the hot plate and textile, in series with the textile itself, and finally the convection+radiation resistance on the topside of the textile sample. Then the series thermal resistances of the textile (from the cut-bar measurements of Fig. 2d for the i-Cool (Cu), and calculated as *d/K* for the uncompressed cotton) and top-side convection plus radiation (as measured from a bare hotplate) are subtracted to get an estimate for $1/h_{ti}$. Thus, this approach to experimentally estimating h_{ti} implicitly assumes that all three measurements (bare hot plate, i-

Cool (Cu), and cotton) all have the same emissivity on their top surfaces. Overall, it must be noted that these estimates of h_{ti} may carry uncertainties. But the uncertainties will not influence our conclusion significantly. Furthermore, h_{ti} is also dependent on the thickness of microclimate, d_m, which is very difficult to assess accurately. Ideally, h_{ti} should be measured between the skin and the fabric which will automatically account for the impact of the thickness of the microclimate on h_{ti}, however here we have assumed it to be same as that obtained from hot plate and cut bar measurement.

The inner surface of the textile is determined by:

$$d_2 C_{\mathrm{v}} \frac{\partial T_2}{\partial t} = \lambda_{\mathrm{v}} w_{1,\mathrm{in}} d_2 \varepsilon_{\mathrm{f}} \frac{\partial C_{\mathrm{f}_2}}{\partial t} + \lambda_{\mathrm{l}} w_{2,\mathrm{in}} d_2 \varepsilon_{\mathrm{f}} \frac{\partial C_{\mathrm{f2}}}{\partial t} - K \frac{T_2 - T_1}{d} + h_{\mathrm{ti}} (T_{\mathrm{s}} - T_2) \tag{13}$$

λ_{v} is the heat of sorption of water vapor by fiber. λ_{l} is a heat of sorption of liquid water by fiber. w_1 is the proportion of the sorption of water vapor by fibers, while w_2 is the proportion of the sorption of liquid water by fibers. ε_{f} is the volume fraction of fibers. d_2 is the thickness of the inner part the textile. C_{v} is volumetric specific heat of the textile. C_{f2} is the water vapor concentration in the fibers at the inner surface of the fabric. K is the thermal conductivity of the textile.

The outer surface of the textile is determined by:

$$d_1 C_{\mathrm{v}} \frac{\partial T_1}{\partial t} = \lambda_{\mathrm{v}} w_{1,\mathrm{out}} d_1 \varepsilon_{\mathrm{f}} \frac{\partial C_{\mathrm{f}_1}}{\partial t} + \lambda_{\mathrm{l}} w_{2,\mathrm{out}} d_1 \varepsilon_{\mathrm{f}} \frac{\partial C_{\mathrm{f1}}}{\partial t} + K \frac{T_2 - T_1}{d} - (C + R)(T_1 - T_{\mathrm{a}}) \tag{14}$$

d_1 is the thickness of the outer part the textile. C is the convective heat transfer coefficient from the textile to the surrounding air. R is the radiative heat transfer coefficient from the textile to the surroundings. Here we assume the R values for the i-Cool and cotton are the same and adopted the value in previous research[16], considering the fact that the emissivity of the textiles can be modified by liquid water during perspiration. In the extreme case (dry textiles), the ~ 0.4 emissivity difference between i-Cool (Cu) and cotton will not influence our final conclusion (< 0.2 °C change for temperature difference between i-Cool (Cu) and cotton).

For the moisture transfer part, starting from the microclimate region between the skin and the inner surface of textile, the water vapor concentration in the microclimate C_{m} is determined by:

$$d_{\mathrm{m}}\frac{\partial C_{\mathrm{m}}}{\partial t}=\frac{1}{\lambda_{\mathrm{v}}}(\alpha\cdot L\cdot\mathrm{regsw}+E_{\mathrm{diff}}(T_{\mathrm{s}},P_{\mathrm{a}}))-h_{\mathrm{ma}}(C_{\mathrm{m}}-C_{\mathrm{a2}}) \quad (15)$$

Where d_{m} is the thickness of the microclimate, and h_{ma} is the mass transfer coefficient from the microclimate to the inner surface of textiles. We take d_{m} ≈10 μm, representing very close contact between the textile and the skin. The water vapor concentration in the air filling the interfibre void space of the textile inner surface C_{a2} can be determined by:

$$d_2\frac{\partial(C_{\mathrm{a2}}\varepsilon_{\mathrm{a2}})}{\partial t}=-d_2w_{1,\mathrm{in}}\varepsilon_{\mathrm{f}}\frac{\partial C_{\mathrm{f2}}}{\partial t}-\frac{D_{\mathrm{a}}}{\tau_{\mathrm{a}}}\varepsilon_{\mathrm{a2}}\frac{C_{\mathrm{a2}}-C_{\mathrm{a1}}}{d}+d_2h_{\mathrm{l\to g}}S_{\mathrm{v}}'[C^*(T_2)-C_{\mathrm{a2}}]+h_{\mathrm{ma}}(C_{\mathrm{m}}-C_{\mathrm{a2}}) \quad (16)$$

The liquid water in the interfibre void space of the textile inner surface can be described as:

$$d_2\rho_{\mathrm{l}}\frac{\partial\varepsilon_{\mathrm{l2}}}{\partial t}=-d_2w_{2,\mathrm{in}}\varepsilon_{\mathrm{f}}\frac{\partial C_{\mathrm{f2}}}{\partial t}-\frac{D_{\mathrm{l}}(\varepsilon_{\mathrm{l2}})}{\tau_{\mathrm{l}}}\cdot\frac{\rho_{\mathrm{l}}\varepsilon_{\mathrm{l2}}-\rho_{\mathrm{l}}\varepsilon_{\mathrm{l1}}}{d}-\frac{a(\varepsilon_{\mathrm{l2}})}{\tau_{\mathrm{l}}}\cdot d_2\cdot\frac{\rho_{\mathrm{l}}\varepsilon_{\mathrm{l2}}-\rho_{\mathrm{l}}\varepsilon_{\mathrm{l1}}}{d}-d_2h_{\mathrm{l\to g}}S_{\mathrm{v}}'[C^*(T_2)-C_{\mathrm{a2}}]+\frac{1}{\lambda_{\mathrm{v}}}((1-\alpha)\cdot L\cdot\mathrm{regsw}) \quad (17)$$

Where $\varepsilon_{\mathrm{f}}+\varepsilon_{\mathrm{a2}}+\varepsilon_{\mathrm{l2}}=1$. D_{a} is the diffusion coefficient of water vapour. τ_{a} is the effective tortuosity of fabric for water vapor. ρ_{l} is density of liquid water. D_{l} is the diffusion coefficient of liquid water in the fabric. τ_{l} is the effective tortuosity of fabric for liquid water. The water vapor concentration in the air filling the interfibre void space of the textile outer surface C_{a1} can be determined by:

$$d_1\frac{\partial(\varepsilon_{\mathrm{a1}}C_{\mathrm{a1}})}{\partial t}=\frac{D_{\mathrm{a}}}{\tau_{\mathrm{a}}}\varepsilon_{\mathrm{a1}}\frac{C_{\mathrm{a2}}-C_{\mathrm{a1}}}{d}-d_1w_{1,\mathrm{out}}\varepsilon_{\mathrm{f}}\frac{\partial C_{\mathrm{f_1}}}{\partial t}+d_1h_{\mathrm{l\to g}}S_{\mathrm{v}}'[C^*(T_1)-C_{\mathrm{a1}}]-h_{\mathrm{c}}(C_{\mathrm{a1}}-C_{\mathrm{ab}}) \quad (18)$$

The liquid water in the interfibre void space of the textile outer surface can be described as:

$$d_1\rho_{\mathrm{l}}\frac{\partial\varepsilon_{\mathrm{l1}}}{\partial t}=-d_1w_{2,\mathrm{out}}\varepsilon_{\mathrm{f}}\frac{\partial C_{\mathrm{f1}}}{\partial t}+\frac{D_{\mathrm{l}}(\varepsilon_{\mathrm{l1}})}{\tau_{\mathrm{l}}}\cdot\frac{\rho_{\mathrm{l}}\varepsilon_{\mathrm{l2}}-\rho_{\mathrm{l}}\varepsilon_{\mathrm{l1}}}{d}+\frac{a(\varepsilon_{\mathrm{l1}})}{\tau_{\mathrm{l}}}\cdot d_1\cdot\frac{\rho_{\mathrm{l}}\varepsilon_{\mathrm{l2}}-\rho_{\mathrm{l}}\varepsilon_{\mathrm{l1}}}{d}-d_1h_{\mathrm{l\to g}}S_{\mathrm{v}}'[C^*(T_1)-C_{\mathrm{a1}}] \quad (19)$$

Where $\varepsilon_{\mathrm{f}}+\varepsilon_{\mathrm{a1}}+\varepsilon_{\mathrm{l1}}=1$. h_{c} is the mass transfer coefficient of water vapor from the outer surface of textiles to the ambient air. $h_{\mathrm{l\to g}}$ is the mass transfer coefficient of liquid water from the fibre surface to the air. C_{ab} is the water vapor concentration in the ambient air. C^* is the saturated water

vapor concentration. A more detailed information of the parameters is summarized in the table of Supplementary Dataset 1 (Definition and details for parameters in modeling)[12, 16-23].

Combining the coupled heat and mass transfer model Eqs. (11)-(19), we can calculate the thermal impacts of different textiles on human body core temperature and skin temperature, under the condition of intense exercise. Here, we assume the initial temperatures of T_c and T_s are 37 °C and 34 °C, respectively. T_a is set as 22 °C.

Supplementary Fig. 23b shows the simulated temperatures with the i-Cool textile and cotton textile, respectively. The i-Cool textile exhibits enhanced evaporation ability and sweat evaporative cooling efficiency, compared to conventional textiles. Hence, we used α = 0.9, η = 0.85 for i-Cool, and α = 0.75, η = 0.4 for cotton. The body core and skin temperatures with the i-Cool textile are approximately 36.7 °C, 38.5 °C, respectively. On the other hand, the body core and skin temperatures of the case with cotton are 38.7 °C, 40.4 °C, respectively.

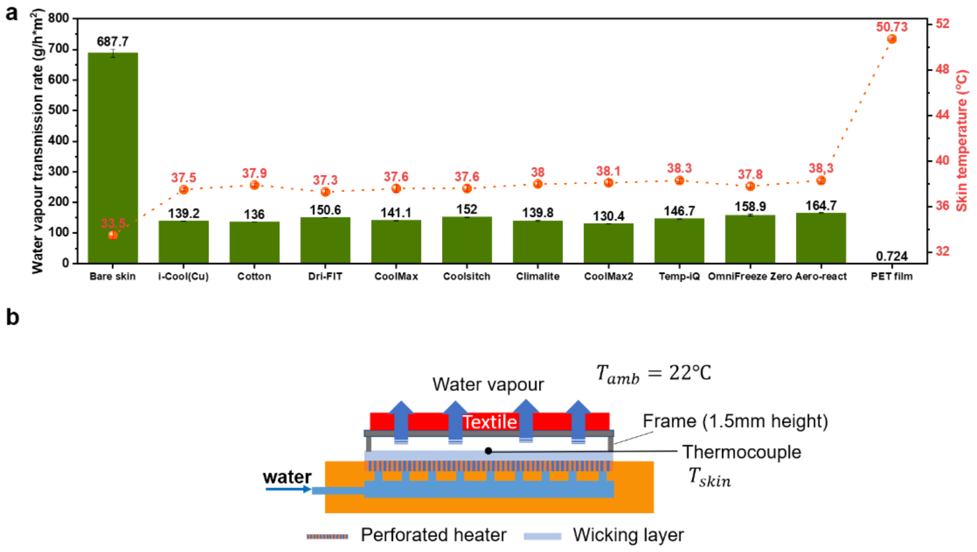

Supplementary Figure 1. a, Water vapour transmission rate of textile samples, bare skin (no textile) and a PET film. The i-Cool (Cu) textile shows comparable water vapour transmission rate with other commercial textiles. Water vapour transmission rate is overall correlated with the cooling effect of the skin with different textiles in the water vapour thermal measurement. The orange dots show the measured skin temperature in the water vapour thermal measurement. **b,** Schematic of the test apparatus of the water vapour thermal measurement (not to scale, in which only water vapour passes through the textile samples. All the error bars represent standard deviation of measured data.

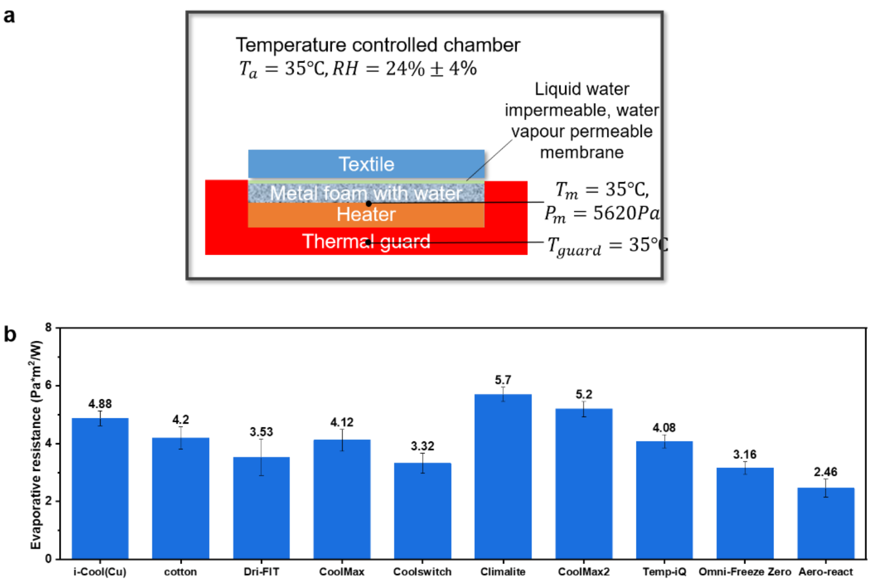

Supplementary Figure 2. Evaporative resistance measurement of different textile samples. a, Schematic of the test apparatus (not to scale), which is according to ASTM 1868-17/ISO11092:2014 with modification. **b,** Evaporative resistance measurement results of various textile samples. All the error bars represent standard deviation of measured data.

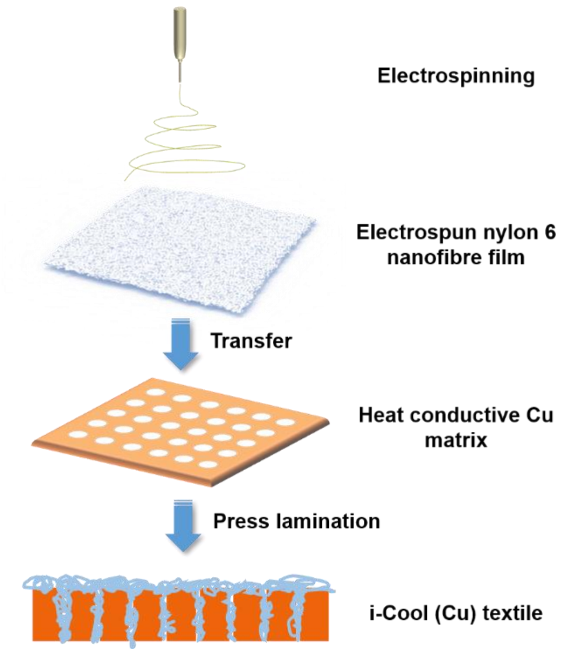

Supplementary Figure 3. Schematic of the fabrication process of the i-Cool (Cu) textile.

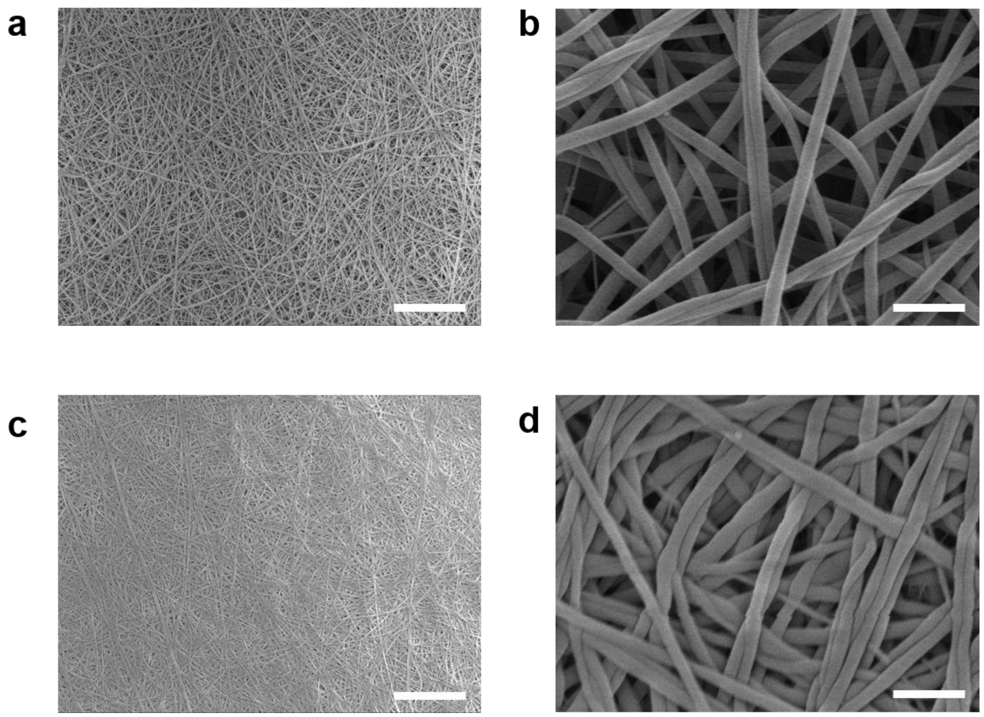

Supplementary Figure 4. SEM images of nylon 6 nanofibres. a, b, Nanofibres in the holes of the Cu heat conductive matrix. **c, d,** Nanofibres on the skeleton of the Cu heat conductive matrix. Scale bars in (**a**) and (**c**), 10 µm. Scale bars in (**b**) and (**d**), 1 µm.

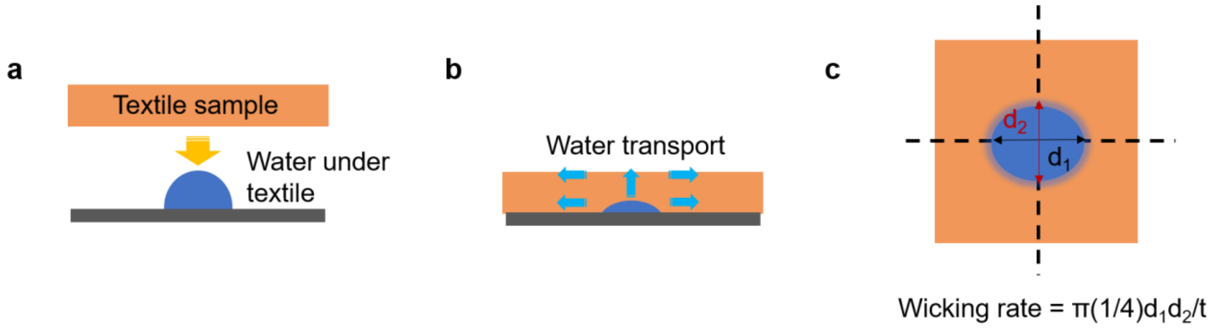

Supplementary Figure 5. Schematic of wicking performance test method. a, A certain amount of water was placed on a platform, and the textile sample was covered on it immediately. **b,** The water was transported in the textile in both horizonal and vertical directions. **c,** The time of water reaching a certain distance on the top surface was recorded and wicking rate was calculated using wicking area divided by wicking time.

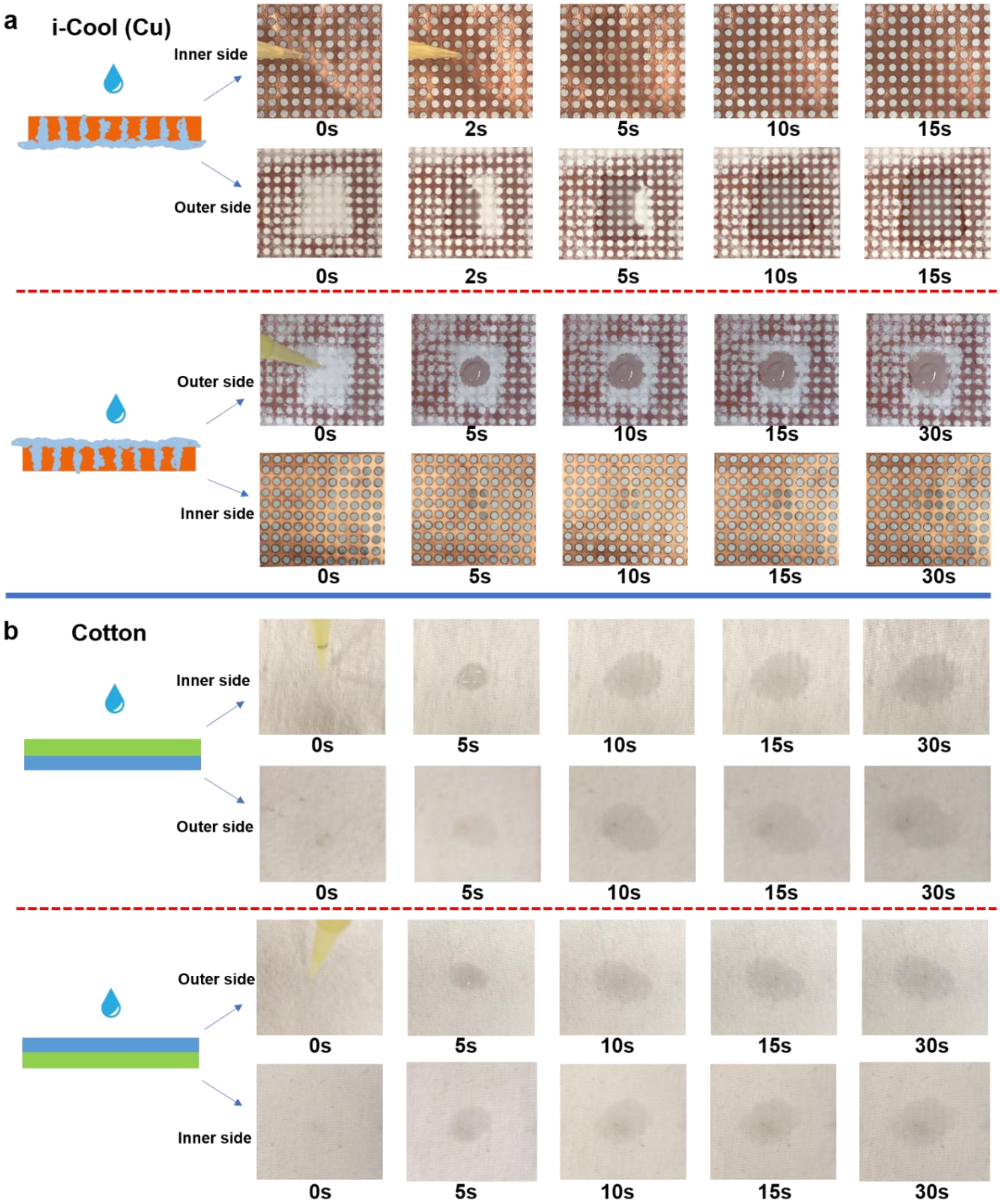

Supplementary Figure 6. Water transport behavior (water droplet, 20 μL) on outer side and inner side of i-Cool (Cu) and cotton when water droplet was added from the outer side and inner side, respectively. Outer side is the side of fabric that is designed to be exposed to air, while inner side is the one exposed to skin. The green and blue colors of cotton fabric schematic just label the two sides of cotton, without other meaning.

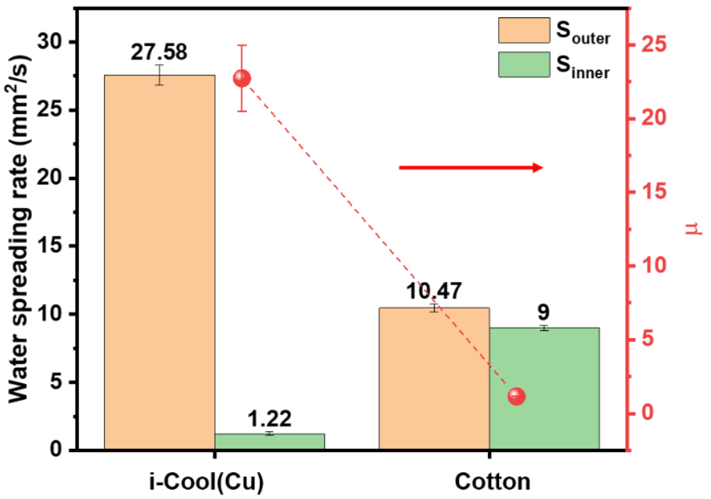

Supplementary Figure 7. Water spreading rate on the outer side and inner side, and the one-way transport index (μ) for i-Cool (Cu) and cotton. All the error bars represent standard deviation of measured data.

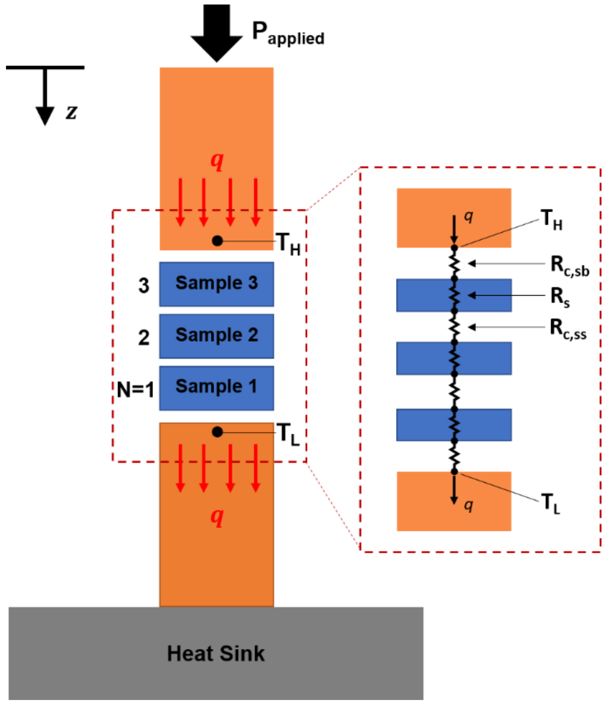

Supplementary Figure 8. Schematic of the cut bar apparatus used in the thermal resistance measurement. Heat flux *q* flows down the upper copper reference bar and through the stacked samples down through the bottom copper reference bar and is dissipated into the aluminum base

heat sink. Each copper reference bar has four thermocouples inserted into the center of the bar, spaced by a pitch of 0.55 inch along the bar. The temperature readings of the thermocouples are used to determine the heat flux q and the temperature drop across the samples ΔT_s, which are required to calculate the total thermal resistance. The red dash box shows the three types of constituent resistances in series in the total thermal resistance measured by the cut bar apparatus when samples are stacked. R_s represents the thermal resistance of a single sample sheet, $R_{c,\ ss}$ represents the thermal contact resistance between adjacent samples, and $R_{c,sb}$ represents the thermal contact resistance between a sample and a copper reference bar.

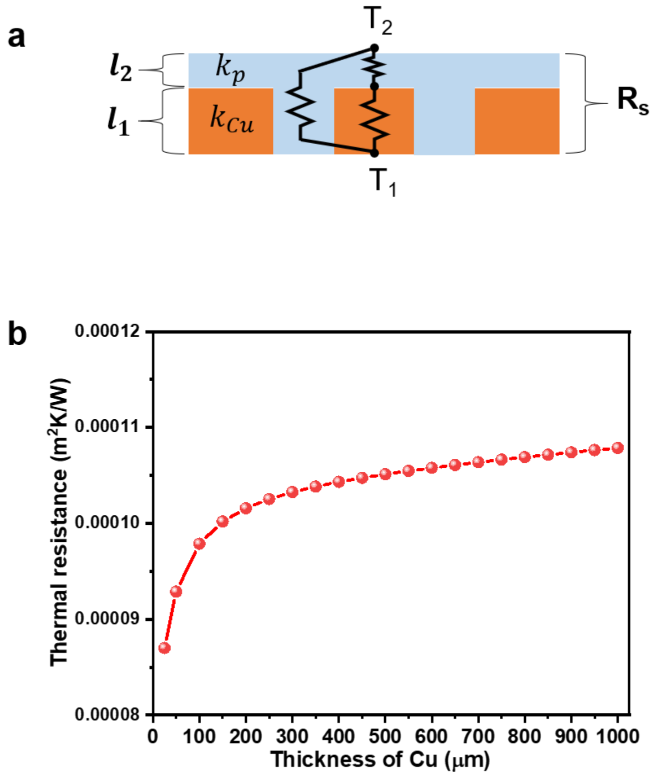

Supplementary Figure 9. Thermal resistor model of i-Cool (Cu) textile. a, Diagram of the series and parallel resistance network proposed in the model of the i-Cool (Cu) thermal resistance. In this schematic, the light blue section represents the nylon 6 nanofibres and the orange section represents the copper metal matrix. **b,** Simulated results of i-Cool (Cu) thermal resistance varying the thickness of Cu.

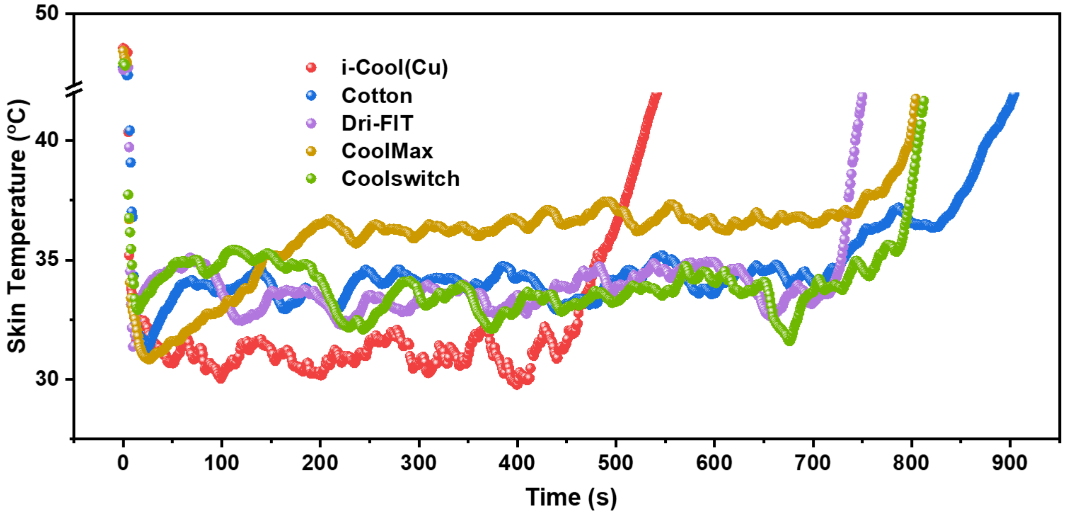

Supplementary Figure 10. A typical set of curves of skin temperature versus time for the i-Cool (Cu) textile and the conventional textiles during evaporation with the same initial water amount (0.1 mL) and skin power density (422.5 W/m^2). The i-Cool (Cu) textile shows much shorter evaporation time and lower skin temperature.

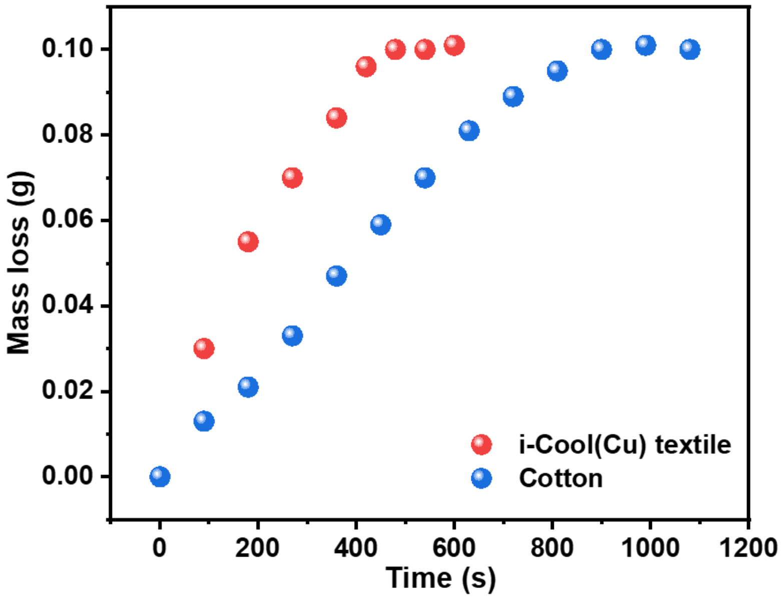

Supplementary Figure 11. Water mass loss versus time during evaporation with i-Cool (Cu) textile and cotton.

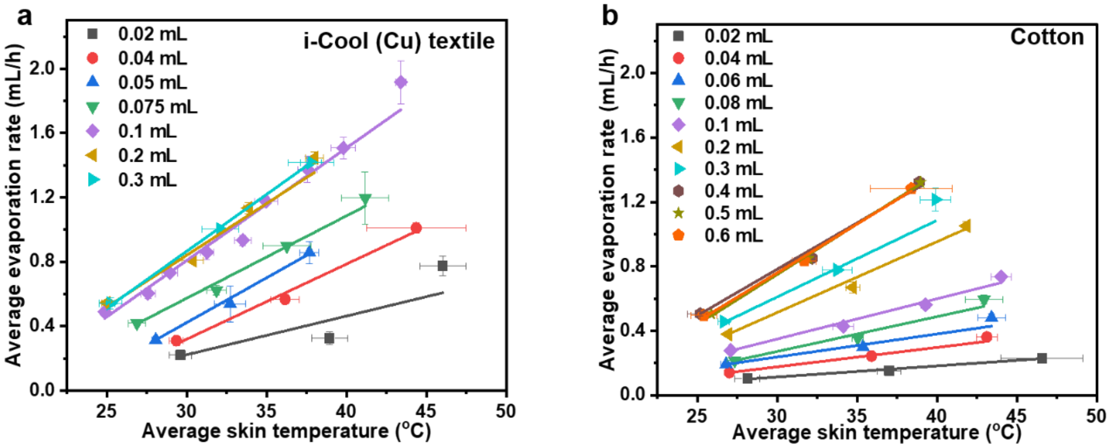

Supplementary Figure 12. Summarized average evaporation rate of i-Cool (Cu) textile **(a)** and cotton **(b)** with various initial water amount and average skin temperature during evaporation. All the error bars represent standard deviation of measured data.

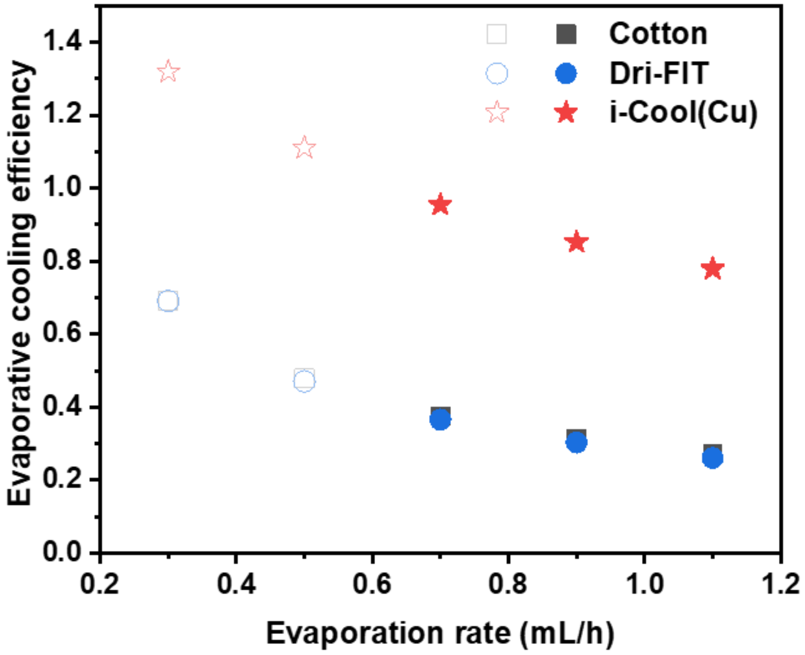

Supplementary Figure 13. Estimated sweat evaporative cooling efficiency of i-Cool (Cu), cotton and Dri-FIT based on the steady-state evaporation rate. The estimated values at higher evaporation rates are more reliable.

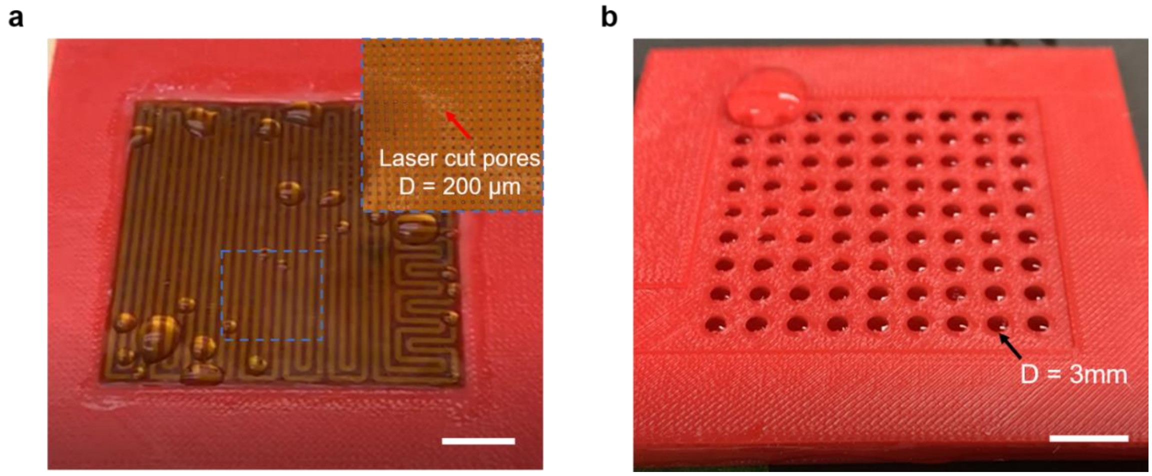

Supplementary Figure 14. Photographs of water outflow situation for perforated flexible polyimide heater with pores of 200 μm in diameter (**a**) and 3D printed acrylonitrile butadiene styrene (ABS) part with holes of 3 mm in diameter (**b**). For both of them, water outflow cannot be uniform. Scale bars, 1 cm. Inset of (**a**) shows the magnified photograph of the perforated heater.

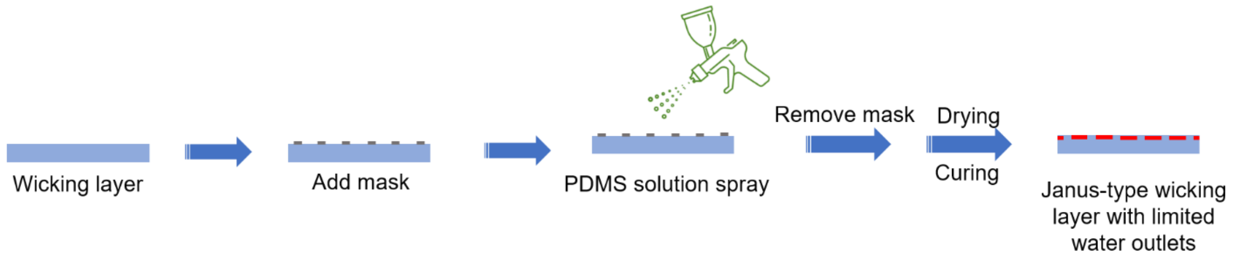

Supplementary Figure 15. Schematic of the fabrication process of the Janus-type wicking layer with limited water outlets.

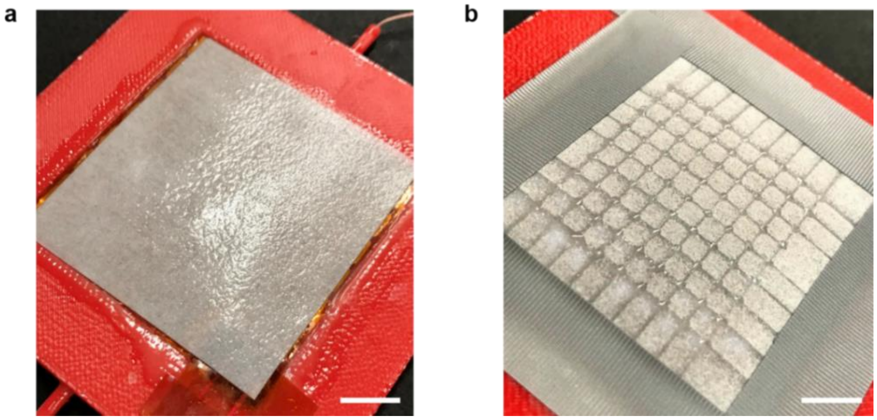

Supplementary Figure 16. Comparison of normal wicking layer and the Janus-type wicking layer with limited water outlets. a, Normal wicking layer can spread water totally inside itself. The surface of it is wet everywhere. **b,** The Janus-type wicking layer with limited water outlets confines water outflow into only the manufactures "sweat spots", mimicking human body sweating situation. Scale bars, 1 cm.

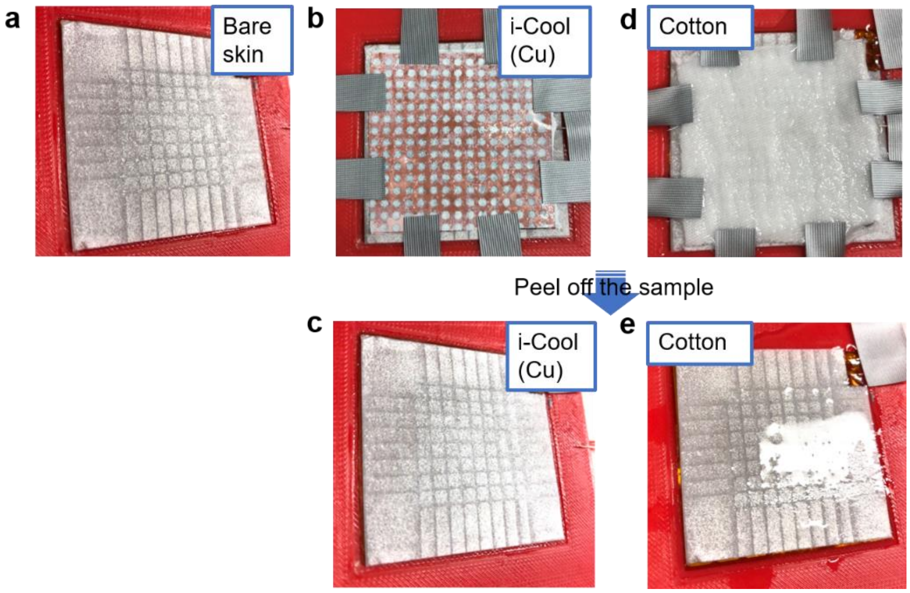

Supplementary Figure 17. Photographs of different samples on the sweating skin platform after a stabilization of 30 minutes, with the same skin temperature and power density, while the sweating rate for different samples was varied to keep the same skin temperature. Obviously, bare skin and the i-Cool (Cu) textile can keep skin cool using sweat much more efficiently than cotton.

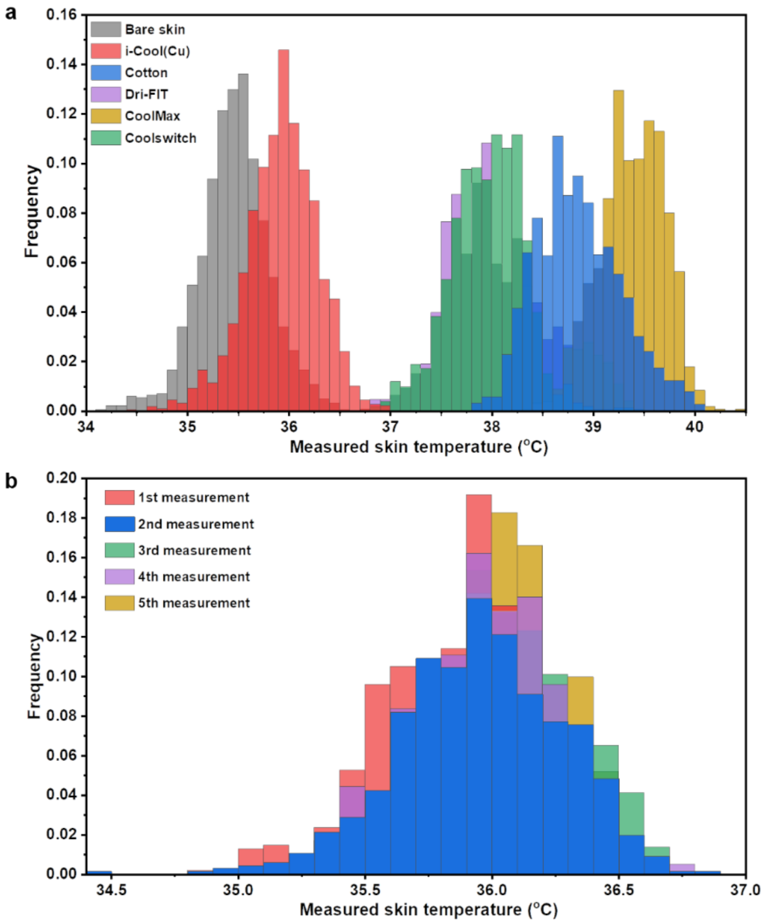

Supplementary Figure 18. Histograms showing data distribution in the artificial sweating skin test. **a,** Histograms of measured skin temperature with different textile samples. **b,** Histograms of measured skin temperature with the i-Cool (Cu) textile for multiple tests

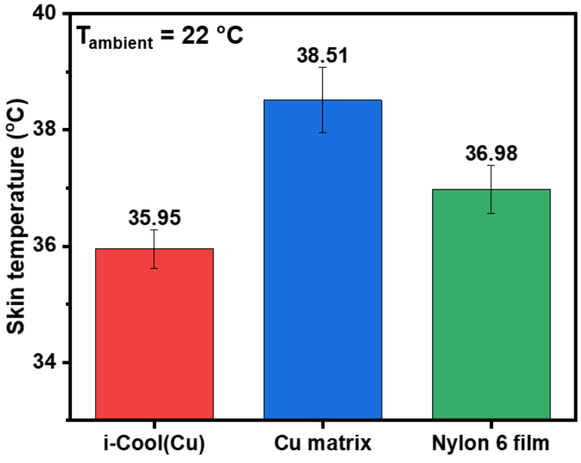

Supplementary Figure 19. Artificial sweating skin test for i-Cool (Cu) textile, Cu matrix and nylon 6 nanofibre film. The separate thermally conductive component and water transport component cannot show similar cooling effect to that of the i-Cool functional structure design. All the error bars represent standard deviation of measured data.

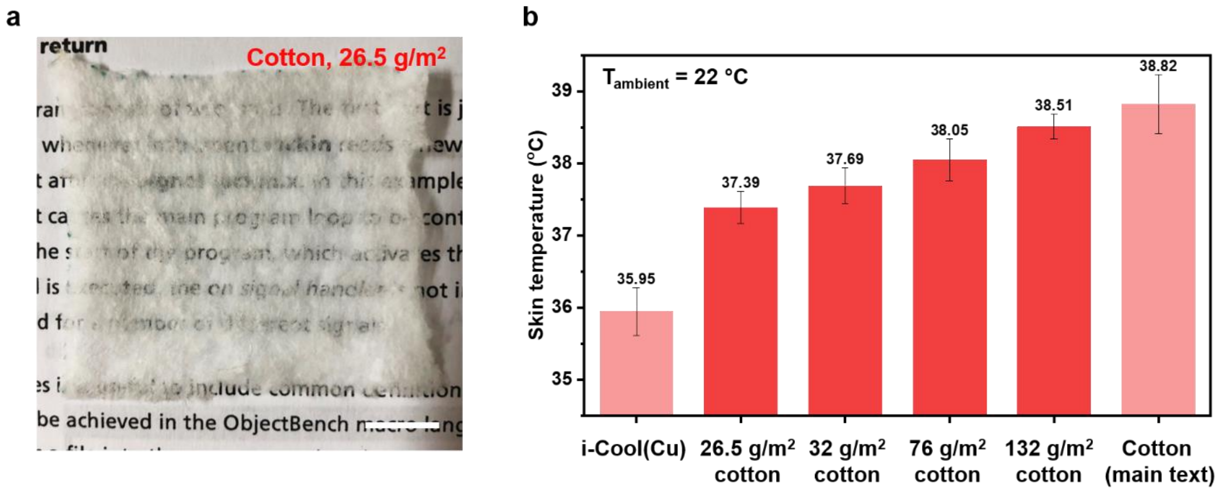

Supplementary Figure 20. Artificial sweating skin test for cotton of various area mass density. **a,** Photograph of the thin cotton sample (area mass density: 26.5 g/m^2). The object behind it can be clearly seen, indicating its insufficient opacity for practical use. Scale bar, 1 cm. **b,** Measurement results comparing i-Cool (Cu) textile, the cotton textile in the main text (~ 135 g/m^2) and cotton samples of various area mass density. All the error bars represent standard deviation of measured data.

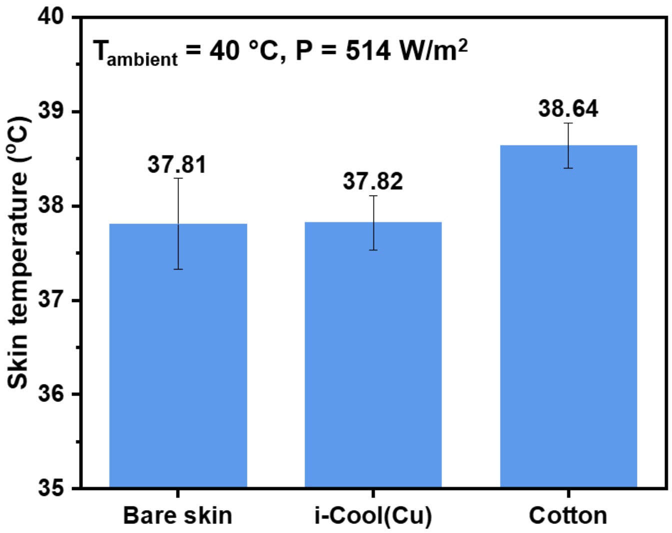

Supplementary Figure 21. Artificial sweating skin test for bare skin, i-Cool (Cu) textile and cotton at high ambient temperature (40 °C). The skin power density used here was adjusted to make the skin temperature for samples lower than the ambient temperature to see if the good heat conduction capability of Cu would cause a reverse effect. All the error bars represent standard deviation of measured data.

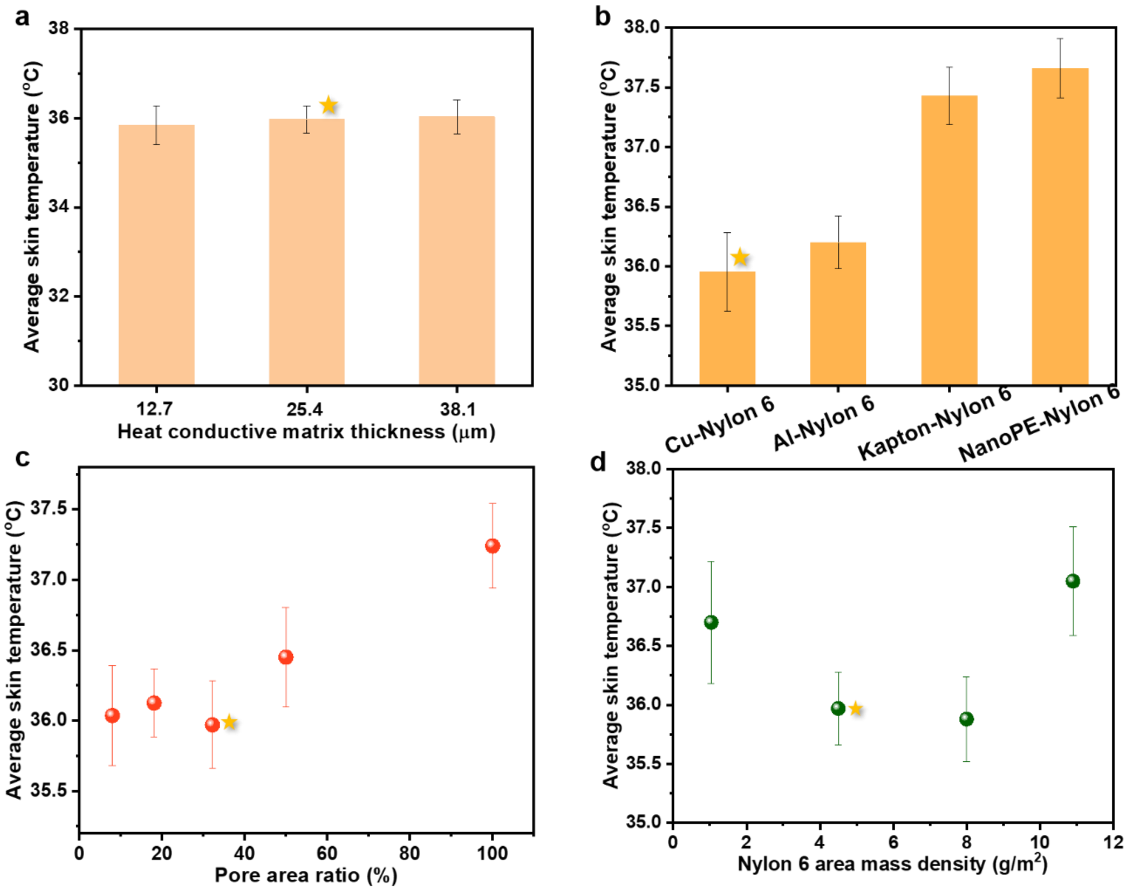

Supplementary Figure 22. Graphs showing how structure parameters of the i-Cool (Cu) influence the cooling performance. a, Experimental results of samples of three different Cu matrix thickness. **b,** Experimental results of samples with different heat conductive matrix materials. **c,** Average skin temperature for i-Cool (Cu) textile samples with different pore area ratio. **d,** Average skin temperature for i-Cool (Cu) textile samples with different nylon 6 nanofibre film area mass density. The star labels in the graphs mean the benchmark i-Cool (Cu) textile sample. All the error bars represent standard deviation of measured data.

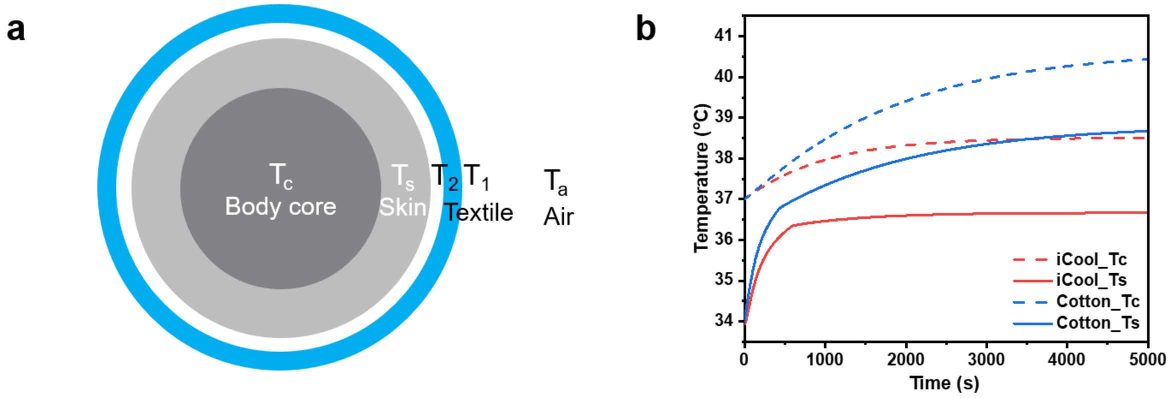

Supplementary Figure 23. Thermal simulation for human body. a, Schematics of the human-clothing-environment system. T_c, T_s, T_2, T_1, T_a represents the temperatures of body core, skin, inner surface of textiles, and outer surface of textiles, respectively. **b,** Simulated core temperature and skin temperature when wearing i-Cool and cotton, respectively. For i-Cool, the set α = 0.9, η = 0.85; for cotton, α = 0.75, η = 0.4.

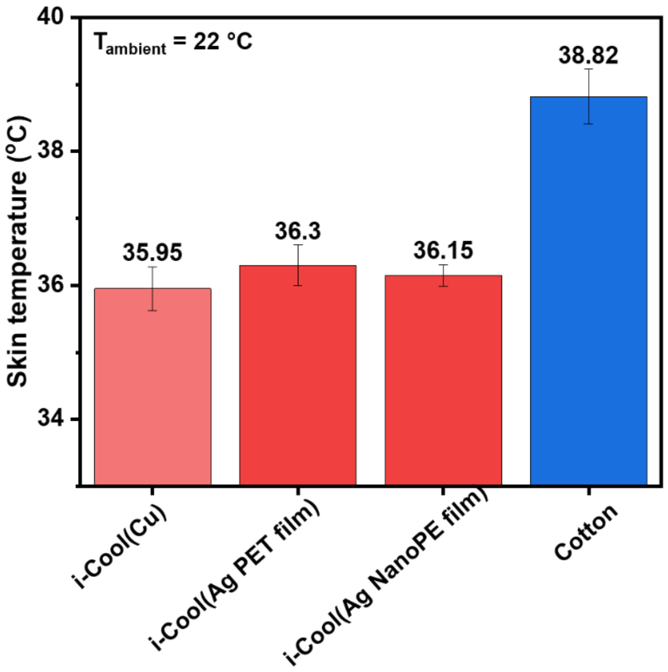

Supplementary Figure 24. Artificial sweating skin test for i-Cool (Cu) textile, i-Cool (Ag PET) textile and i-Cool (Ag NanoPE) textile and cotton. All the error bars represent standard deviation of measured data.

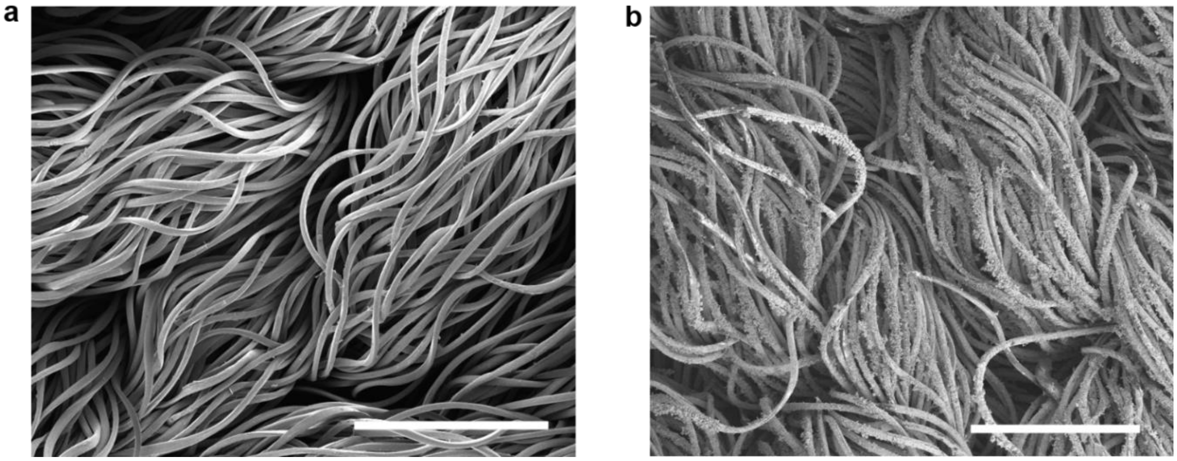

Supplementary Figure 25. a, SEM image of the original Dri-FIT fabric made of PET fibres. **b,** The Ag coated Dri-FIT fabric. Scale bars, 300 µm.

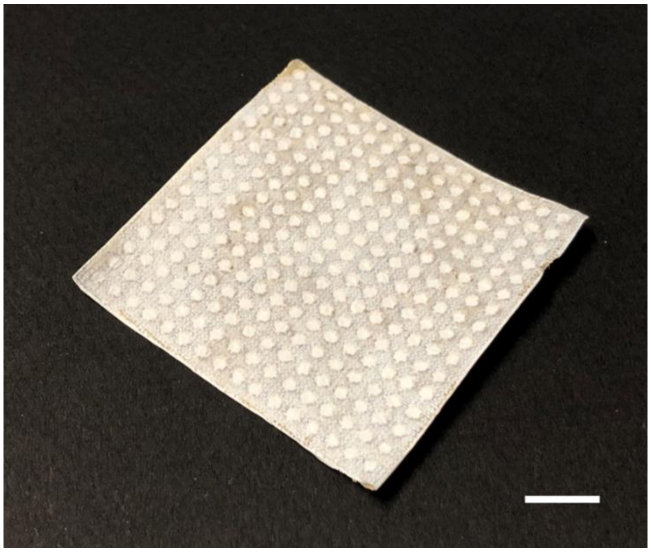

Supplementary Figure 26. Photograph of the i-Cool (Ag) textile based on CoolMax as the substrate. Scale bar, 1 cm.

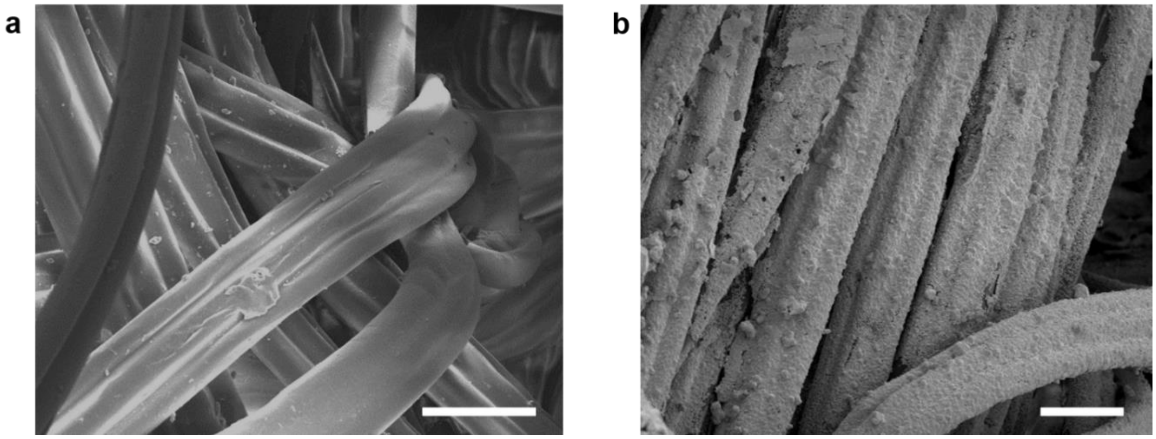

Supplementary Figure 27. a, SEM image of the original CoolMax fabric made of PET fibres. Scale bar, 20 μm. **b,** SEM image of the Ag coated fibres of CoolMax. Scale bar, 20 μm.

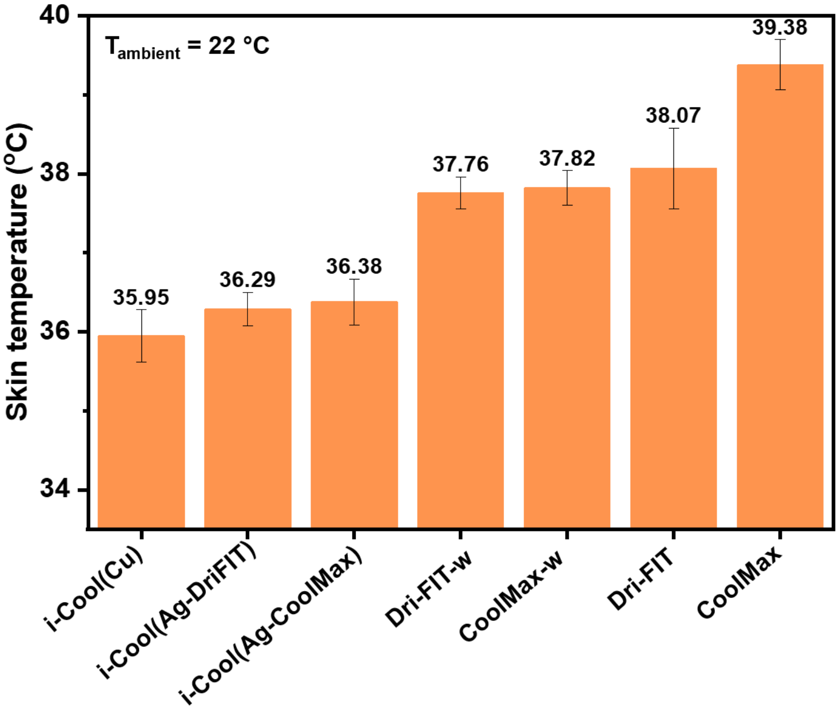

Supplementary Figure 28. Measured skin temperature of i-Cool (Ag) based on Dri-FIT and CoolMax, together with other control samples, in the artificial sweating skin test. The "Dri-FIT-w" and "CoolMax-w" refer to the fabric samples with the same modification as i-Cool (Ag) samples except the Ag coating. All the error bars represent standard deviation of measured data.

References:

1. Lomax, G. R. Breathable polyurethane membranes for textile and related industries. *J. Mater. Chem.* **17**, 2775-2784 (2007)
2. Hunter, L., Fan, J. Chapter 11: Waterproofing and breathability of fabrics and garments (Woodhead Publishing Series in Textiles, 2009)
3. Kuno, Y. Human perspiration, Chapter I, 21-28 (Springfield, 1956)
4. Hardy, J. D. & Dubois, E. F. Regulation of heat loss from the human body. *P. Natl. A. Sci.* **23**, 624-631 (1937).
5. G. Song, S. Mandal. Chapter 3: Testing and evaluating the thermal comfort of clothing ensembles. *Performance Testing of Textiles* (Woodhead Publishing Series in Textiles, 2016)
6. Saums, D. ASTM D 5470-06 Thermal Interface Material Test Stand (DS&A LLC, 2006).
7. Lubner, S. D., Kaur, S., Fu, Y., Battaglia, V., & Prasher, R. S. Identification and characterization of the dominant thermal resistance in lithium-ion batteries using operando 3-omega sensors. *J. Appl. Phy*. **127**, 105104 (2020).
8. Incropera, F. P., Lavine, A. S., Bergman, T. L., & DeWitt, D. P. Fundamentals of heat and mass transfer. (Wiley, 2007)
9. Zhou, H., Zhang, S., & Yang, M. The thermal conductivity of nylon 6/clay nanocomposites. *Journal of applied polymer science* **108**, 3822-3827 (2008).
10. https://www.m-ep.co.jp/en/pdf/product/iupi_nova/physicality_04.pdf
11. http://www.matweb.com/search/DataSheet.aspx?MatGUID=8d78f3cfcb6f49d595896ce6ce6a2ef1&ckck=1 (0.23-0.342)
12. Gagge, A. P., Fobelets, A. P., Berglund, L. G. A standard predictive index of human response to the thermal environment. *ASHARE Trans.* **1**, 709-731 (1986).
13. Craig, F. N. & Moffitt, J. T. Efficiency of evaporative cooling from wet clothing. *J. Appl. Physiol.* **36**, 313–316 (1974).
14. Havenith, G. *et al.* Evaporative cooling: Effective latent heat of evaporation in relation to evaporation distance from the skin. *J. Appl. Physiol.* **114**, 778–785 (2013).
15. Guan, M. *et al.* Apparent evaporative cooling efficiency in clothing with continuous perspiration: A sweating manikin study. *Int. J. Therm. Sci.* **137**, 446–455 (2019).

16. Li, Y. and Holcombe, B.V., Mathematical Simulation of Heat and Mass Transfer in a Human Clothing Environment, *Text. Res. J.* **67**, 389-397 (1998).
17. Li, B. et al. A simplified thermoregulation model of the human body in warm conditions. *Appl. Ergon.* **59**, 387-400 (2017).
18. Fu G. A transient, 3-D mathematical thermal model for the clothed human. PhD thesis, Kansas State Univerisity, Kansas (1995).
19. Smith CE. A transient, three-dimensional model of the human thermal system. PhD thesis, Kansas State University, Kansas (1991).
20. Zhu, Q. Y. & Li, Y. A model of coupled liquid moisture and heat transfer in porous textiles with consideration of gravity. Numerical Heat Transfer, Part A, 43, 501 (2003).
21. Buck Research CR-1A User's Manual, Appendix 1. (PDF) (1996)
22. Abbas, A. et al. Improving Thermal Conductivity of Cotton Fabrics Using Composite Coatings Containing Graphene, Multiwall Carbon Nanotube or Boron Nitride Fine Particles. *Fibers and Polymers*, **14**, 1641-1649 (2013).
23. https://www.engineeringtoolbox.com/air-diffusion-coefficient-gas-mixture-temperature-d_2010.html
