# Supplementary material for: Integrated cooling (i-Cool) textile of heat conduction and sweat transportation for personal perspiration management
Source: Nat Commun. 2021 Oct 21;12:6122. doi: 10.1038/s41467-021-26384-8 (PMC8531342; doi:10.1038/s41467-021-26384-8)
Supplement: Supplementary file 3 — Description of Additional Supplementary Files [file 41467_2021_26384_MOESM3_ESM.docx]

**Description of Additional Supplementary Files**

**File Name:** Supplementary Data 1

**Description:** Definition and details for parameters in the modelling.
