# Supplementary material for: Integrated cooling (i-Cool) textile of heat conduction and sweat transportation for personal perspiration management
Source: Nat Commun. 2021 Oct 21;12:6122. doi: 10.1038/s41467-021-26384-8 (PMC8531342; doi:10.1038/s41467-021-26384-8)
Supplement: Supplementary file 4 — Supplementary Dataset 1 [file 41467_2021_26384_MOESM4_ESM.zip › Supplementary Data file (modelling parameter definition).pdf]

Symbol	Definition	Expression / Value	Unit
m_c	mass of the body core	70 [ref.12]	kg
A	area of the body core	1.8 [ref.12]	m^2
C_c	specific heat of body core	2968 [ref.17]	$J \cdot kg^{-1} \cdot C^{-1}$
$M(T_s, T_c)$	metabolic heat production	$M(T_s, T_c) = M_0 + 19.4 * \text{Colds} * \text{Coldc}$<br>$\text{Colds} = \max(T_{s0} - T_s, 0)$<br>$\text{Coldc} = \max(T_{c0} - T_c, 0)$<br>$M_0$ = 400 $W \cdot m^{-2}$, T_{s0} = 33.7 °C, T_{c0} = 36.8 °C<br>[ref.12, 18]	$W \cdot m^{-2}$
$C_{res}(M, T_a)$	convective heat loss from respiration	$C_{res}(M, T_a) = 0.0014 * M * (34 - T_a)$<br>[ref.17]	$W \cdot m^{-2}$
$E_{res}(M, T_a)$	evaporative heat loss from respiration	$E_{res}(M, T_a) = 0.0000173 * M * (5867 - P_a)$<br>[ref.17]	$W \cdot m^{-2}$
$H_b(T_c, T_s)$	heat transfer from body core to skin via blood circulation	$H_b(T_c, T_s) = (K_{min} + \text{skbfu} * \text{rb} * \text{cb}) * (T_c - T_s)$<br>$\text{skbfu} = 0.0278 * \text{skbf}/10^5$<br>$\text{skbf} = (6.3 + 200 * \text{Warmc})/(1 + 0.1 * \text{Colds}) \leq \text{skbf} \leq 90$<br>$\text{rb} = 1000$<br>$\text{cb} = 3760$<br>[ref.12, 17]	$W \cdot m^{-2}$
d_s	thickness of the skin layer	1<br>[ref.17]	mm
ρ_s	density of the skin layer	1000<br>[ref.17]	$kg \cdot m^{-3}$

C_s	specific heat of the skin layer	3760 [ref.17]	$J \cdot kg^{-1} \cdot C^{-1}$
$E_{sw}(T_c, T_s)$	heat loss by sweat evaporation	$\alpha * \eta * L * \text{regsw}$<br>$\text{regsw} = 170 * \text{warmb} * exp(\frac{\text{warms}}{10.7})$, unit [g/m^2·h]<br>$L = 0.68$, unit [W·h/g]<br>[ref.12]	$W \cdot m^{-2}$
$E_{diff}(T_s, P_a)$	heat loss by water vapor diffusing through the skin layer	$E_{diff}(t) = 0.00305(256T_s - 3373 - P_a)$<br>[ref.17]	$W \cdot m^{-2}$
P_a	water vapour partial pressure	$P_a = 611.21 exp\left(\left(18.67 - \frac{T}{234.5}\right) * \frac{T}{257.14 + T}\right) * \text{RH}$<br>[ref.21]	Pa
RH	Relative humidity	20%	N/A
h_{ti}	combined heat transfer coefficient in the microclimate	362 and 83, based on experiments	$W \cdot m^{-2} \cdot K^{-1}$
d	thickness of the textile	400	µm
d_1, d_2	thickness of the inner and outer part of textile	$d_1 = d_2 = \frac{1}{2} d$	µm
C_v	volumetric specific heat of the textile	1.75 x 10^3 [ref. 16]	$kg \cdot m^{-3} \cdot K^{-1}$
λ_v	heat sorption of water vapor by fiber	2522 [ref. 16, 20]	$KJ \cdot kg^{-1}$
λ_l	heat sorption of liquid water by fiber	2260 [ref. 20]	$KJ \cdot kg^{-1}$

λ	Latent heat of water evaporation	2256	$\mathrm{KJ \cdot kg^{-1}}$
w_1, w_2	Proportion of the sorption of water vapor and liquid water by fibers	$w_1 = \frac{1-\delta}{\varepsilon_\mathrm{a}/\varepsilon}$ $w_2 = 1 - \frac{\varepsilon_\mathrm{a}}{\varepsilon} + \frac{\varepsilon_\mathrm{a}}{\varepsilon}\delta$ $\delta = (\frac{\varepsilon_\mathrm{l}}{\varepsilon})^{0.6}$ [ref. 20]	N/A
K	thermal conductivity of the textile	0.24 (from Fig. 2d) and 0.04 [ref.22]	$\mathrm{W \cdot m^{-1} \cdot K^{-1}}$
C	convective heat transfer coefficient of outer surface to air	2.91 [ref. 16]	$\mathrm{W \cdot m^{-2} \cdot K^{-1}}$
R	radiative heat transfer coefficient of outer surface to air	5.23 [ref. 16]	$\mathrm{W \cdot m^{-2} \cdot K^{-1}}$
d_m	thickness of the microclimate	10	µm
$E_\mathrm{sk}(T_\mathrm{s}, T_\mathrm{c}, P_\mathrm{a})$	total skin evaporation heat loss	$E_\mathrm{sw}(T_\mathrm{c}, T_\mathrm{s}) + E_\mathrm{diff}(T_\mathrm{s}, P_\mathrm{a})$	$\mathrm{W \cdot m^{-2}}$
h_ma	mass transfer coefficient from the microclimate to the inner surface of textiles	3.5 [ref. 16]	$\mathrm{m \cdot s^{-1}}$
$C_\mathrm{f1}, C_\mathrm{f2}$	water vapor concentration in the fibers	$\frac{\partial C_\mathrm{f1}}{\partial t} = h_\mathrm{cf} S_\mathrm{v1}(C_\mathrm{f1} - C_\mathrm{a1})$ $\frac{\partial C_\mathrm{f2}}{\partial t} = h_\mathrm{cf} S_\mathrm{v2}(C_\mathrm{f2} - C_\mathrm{a2})$ [ref. 16]	$\mathrm{kg \cdot m^{-3}}$
$S_\mathrm{v1}, S_\mathrm{v2}$	Specific volume of the fabric	$S_\mathrm{v1} = (2/\varepsilon)(\varepsilon_\mathrm{f}\delta_1/R_\mathrm{f})$ $S_\mathrm{v2} = (2/\varepsilon)(\varepsilon_\mathrm{f}\delta_2/R_\mathrm{f})$ [ref. 20]	$\mathrm{m^{-1}}$

S_v'	Specific volume of the wetting fabric	$\varepsilon_a S_v$ [ref. 20]	m^{-1}
ε	fabric porosity	0.7 $\varepsilon = \varepsilon_a + \varepsilon_l$	N/A
ε_f	Volume fraction of fibers	$\varepsilon + \varepsilon_f = 1$	N/A
ε_a, ε_l	Volume fraction of water vapor and liquid water	$\varepsilon = \varepsilon_a + \varepsilon_l$ $\varepsilon_l(t = 0) = 0.5$ [ref. 20]	N/A
D_a	diffusion coefficient of water vapor in air of the porous textile	0.242×10^{-4} [ref.23]	$m^2 \cdot s^{-1}$
τ_a	effective tortuosity of fabric for water vapor	2	N/A
τ_l	effective tortuosity of fabric for liquid water	2	N/A
ρ_l	Density of the liquid water	1000	$kg \cdot m^{-3}$
D_l	Diffusion coefficient of liquid water in the fabric	$D_l(\varepsilon_l) = \frac{3\sigma \cdot cos\phi \cdot sin^2\beta \cdot d_c \cdot \varepsilon_l}{20\eta\varepsilon}$ σ: surface tension, 31 mN/m ϕ: contact angle β:average angle of the capillaries in fabrics, 20° d_c: largest effective radius of the capillaries, 600 µm η: dynamic viscosity, 8.9×10^{-4} $kg \cdot m^{-1} \cdot s^{-1}$ [ref. 20]	$m^2 \cdot s^{-1}$
$a(\varepsilon_l)$		$a(\varepsilon_l) = \frac{9g \cdot sin^2\beta \cdot \rho_l \cdot d_c^2 \cdot \varepsilon_l^2}{40\eta\varepsilon^2}$	N/A

		g: accelaeration of gravity, 10 m·s^{-2} [ref. 20]	
h_c	Water vapor mass transfer coefficient from the outer surface of textiles to the ambient air	6.4 [ref. 16]	$m \cdot s^{-1}$
$h_{l \to g}$	Liquid water mass transfer coefficient from the fibre surface of textiles to the ambient air	0.137 for cotton<br>0.274 for i-Cool<br>[ref. 20]	$m \cdot s^{-1}$
C^*	Saturated water vapor concentration	$0.0022 * 611.21 * exp\left(\left(18.67 - \frac{T}{234.5}\right) * \frac{T}{257.14 + T}\right)/(T + 273.15)$	$kg \cdot m^{-3}$
C_{ab}	water vapor concentration in the air	$0.02 * RH$	$kg \cdot m^{-3}$

Note: all the reference number listed in this table refers to the reference list in Supplementary Information.
